# Role of Synaptic Structural Plasticity in Impairments of Spatial Learning and Memory Induced by Developmental Lead Exposure in Wistar Rats

**DOI:** 10.1371/journal.pone.0115556

**Published:** 2014-12-23

**Authors:** Yongmei Xiao, Hongjun Fu, Xiaojie Han, Xiaoxia Hu, Huaiyu Gu, Yilin Chen, Qing Wei, Qiansheng Hu

**Affiliations:** 1 Department of Preventive Medicine, School of Public Health, Sun Yat-Sen University, Guangzhou 510080, China; 2 The Jackson Laboratory, 600 Main Street, Bar Harbor, Maine 04609, United States of America; 3 School of Medicine, Sun Yat-Sen University, Guangzhou 510080, China; Northeastern University, United States of America

## Abstract

Lead (Pb) is found to impair cognitive function. Synaptic structural plasticity is considered to be the physiological basis of synaptic functional plasticity and has been recently found to play important roles in learning and memory. To study the effect of Pb on spatial learning and memory at different developmental stages, and its relationship with alterations of synaptic structural plasticity, postnatal rats were randomly divided into three groups: Control; Pre-weaning Pb (Parents were exposed to 2 mM PbCl_2_ 3 weeks before mating until weaning of pups); Post-weaning Pb (Weaned pups were exposed to 2 mM PbCl_2_ for 9 weeks). The spatial learning and memory of rats was measured by Morris water maze (MWM) on PND 85–90. Rat pups in Pre-weaning Pb and Post-weaning Pb groups performed significantly worse than those in Control group (*p*<0.05). However, there was no significant difference in the performance of MWM between the two Pb-exposure groups. Before MWM (PND 84), the number of neurons and synapses significantly decreased in Pre-weaning Pb group, but not in Post-weaning Pb group. After MWM (PND 91), the number of synapses in Pre-weaning Pb group increased significantly, but it was still less than that of Control group (*p*<0.05); the number of synapses in Post-weaning Pb group was also less than that of Control group (*p*<0.05), although the number of synapses has no differences between Post-weaning Pb and Control groups before MWM. In both Pre-weaning Pb and Post-weaning Pb groups, synaptic structural parameters such as thickness of postsynaptic density (PSD), length of synaptic active zone and synaptic curvature increased significantly while width of synaptic cleft decreased significantly compared to Control group (*p*<0.05). Our data demonstrated that both early and late developmental Pb exposure impaired spatial learning and memory as well as synaptic structural plasticity in Wistar rats.

## Introduction

Lead (Pb) is an abundantly existing heavy-metal pollutant in the environment and is a strong toxicant for the development of central nervous system (CNS) in children and animals [1]. Cognitive impairments including various aspects of learning and memory are major clinical symptoms of lead-poisoned children [2]–[5]. Numerous studies have attempted to model this effect of Pb in rats, with the majority of studies focusing on hippocampal-associated spatial learning and memory processes [6], [7]. Developmental Pb exposure has been found to impair learning and memory in animals via affecting the synaptic functional plasticity, i.e. long-term potentiation (LTP) or long-term depression (LTD) [8], [9]. LTP and LTD are typical representations of synaptic functional plasticity and have been considered as physiological models of learning and memory [10], [11].

In addition to synaptic functional plasticity, synaptic structural plasticity is also found to be directly associated with LTP induction and thus plays very important roles in learning and memory [12]. Alterations in the number and size of synapses, synaptic cleft, the thickness of postsynaptic density, the length of synaptic active zone, and synaptic curvature contribute to changes in synaptic structural plasticity, which is closely related to synaptic function and considered to be one physiological base of synaptic functional plasticity [13]. Furthermore, changes in the synaptic function are always accompanied with alterations in the synaptic structure [14]. Chronic developmental low-level Pb exposure (i.e. gestation day 16 to postnatal day 114) significantly reduced the BrdU positive cellsinthe dentate granule cell (DG) layer of adult rat hippocampus at 28 days after the last injection of BrdU although it was not found to impair the spatial learning, indicating that early developmental Pb exposure might affect the neurogenesis and possible other aspects of synaptic structural plasticity in rat hippocampus [15].

In this study, we investigated the effects of low-level Pb exposure at different developmental stages on the synaptic structural plasticity as well as spatial learning and memory in Wistar rats. We found that early developmental Pb exposure (pre-weaning) induced the reduction in the number of neurons and synapses as well as the damage of synaptic structural plasticity, while late developmental Pb exposure (post-weaning) impaired the synaptic structural plasticity without affecting the number of neurons and synapses in rats. Both of Pb exposures before and after weaning impaired the spatial learning and memory in rat pups.

## Materials and Methods

### 1. Ethics Statement

This study was carried out in strict accordance with the recommendations in the Guide for the Care and Use of Laboratory Animals of the National Institutes of Health. The protocol was approved by the Committee on the Ethics of Animal Experiments of the Sun Yat-Sen University, China (Permit Number: 2011-0801). All surgery was performed under urethane anesthesia, and all efforts were made to minimize suffering.

### 2. Animals and Pb exposure protocol

Three-month-old specific pathogen free (SPF) Wistar rats (30 females: 180–200 g; 15 males: 220–240 g) were obtained from the Animal Facility of Southern Medical University (Guangzhou, China). They were housed in controlled conditions of 12-h light: 12-h dark cycle, temperature (23°C), and humidity (60%). Animals were given ad libitumaccess to food and water.

**Figure 1:**
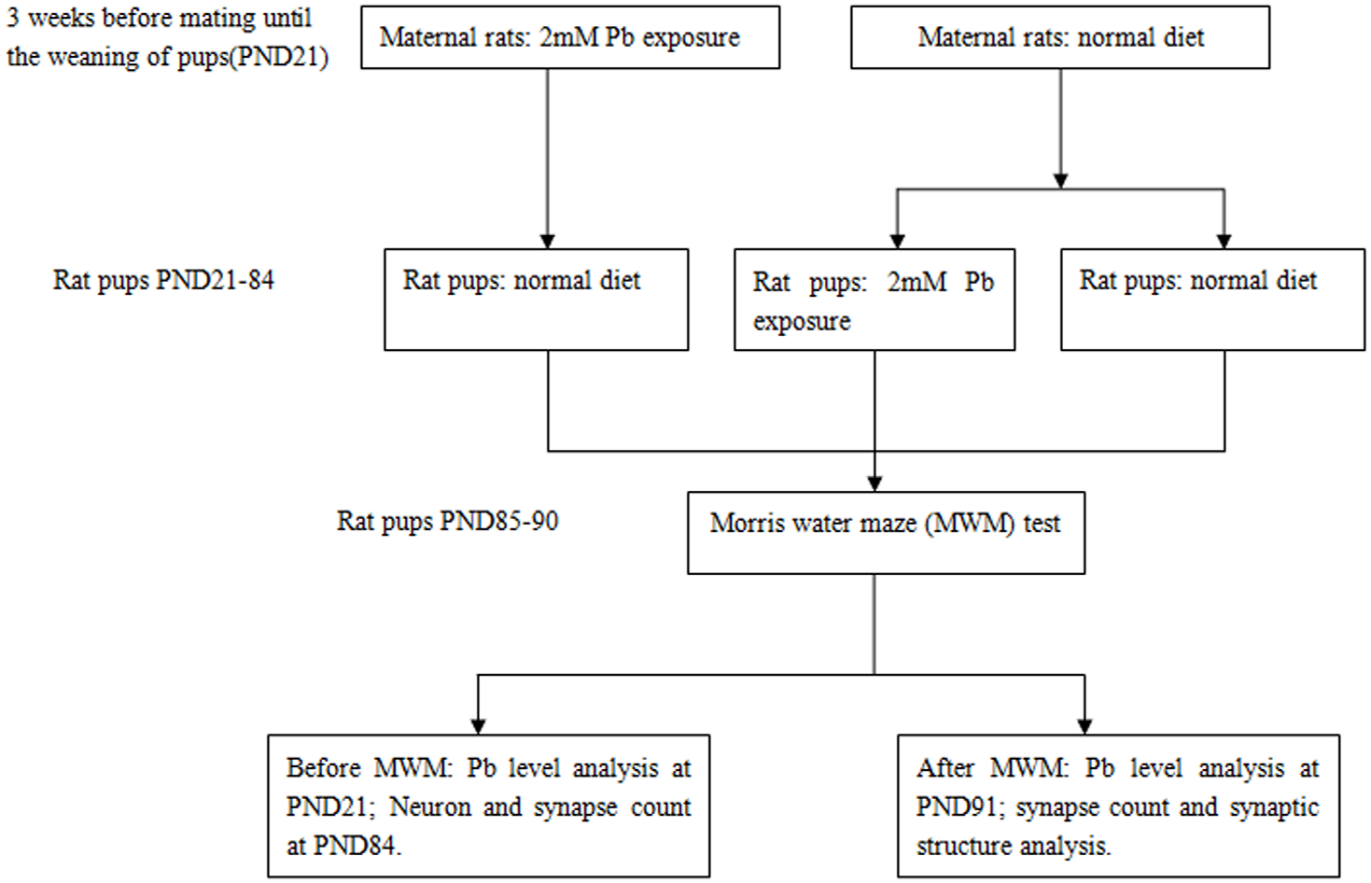
The flowchart depicting exposure protocols and time duration of exposure.

After 1-week normal feeding for accommodation, the female Wistar rats were divided into two groups. 20 female Wistar rats were fed with normal drinking water and the rest 10 females were fed with 2 mM PbCl_2_ via drinking water 3 weeks prior to mating. Three weeks later, the above two groups of female Wistar rats were housed with male Wistar rats at the ratio of 2∶1 and were fed with normal drinking water. The F1 generation pups were weaned on postnatal day 21 (PND 21). After weaning, the rat pups were divided into three groups and each group contained four or five subgroups (1 pup was selected per litter, total 10 pups per subgroup, half male and half female). The three groups are as follows: Control (5 subgroups, Both maternal rats and weaned pups continuously had normal diet); Pre-weaning Pb (5 subgroups, Maternal rats were exposed to Pb 3 weeks before mating until the weaning of pups and weaned pups had normal diet); Post-weaning Pb (4 subgroups, Maternal rats had normal diet and weaned pups were exposed to Pb for 9 weeks). On PND 21, pups of one subgroup in Control and Pre-weaning Pb groups were killed to test Pb concentrations in blood and hippocampus; on PND 91 pups of one subgroup of each group were killed to test Pb concentrations in blood and hippocampus. On PND 84, pups of two subgroups of each group were killed to test the number of neurons and synapses in rat hippocampus, respectively. The rest one subgroup pups were trained with Morris water maze from PND 85 to PND 90 and then subjected to the measurement of the number of synapses and the synaptic structural plasticity in the CA1 region of rat hippocampus. The flowchart depicting exposure protocols and time duration of exposure as Fig. 1 and the experimental assessment timelines for those three groups of rats were described in Table 1.

**Table 1:** The experimental assessment timelines.

Group(subgroup)	PND21-Pb level analysis	PND84-Neuron and synapse count	PND85–90- MWM test	PND91-Pb level analysis, synapse count and synaptic structure analysis
control (5)	√	√	√	√
Pre-weaning Pb (5)	√	√	√	√
Post-weaning Pb (4)		√	√	√

Note: PND = postnatal day; MWM = Morris water maze; Pb = 2 mM PbCl_2_. The symbol of √ indicates that the specific assessment was performed on that day.

The dosage of Pb was chosen according to our previous publication [16] and pre-test results. Furthermore, we found this dosage of Pb exposure in Wistar rats could result in blood Pb level of ∼100 µg/L, which is considered as the lower bound threshold for Pb neurotoxicity in children [1]. The amount of water in the presence or absence of Pb consumed by each rat and its body weight was measured each day. There was no significant difference in consuming pure water or Pb-containing water proportional to body weight among different groups of rats (data not shown). The rats were sacrificed by being anesthetized with urethane (25%, 0.5 ml/100 g, i.p.) and followed by bilateral thoracotomy.

### 3. Morris Water maze (MWM) test

The spatial learning and memory test was assessed in the MWM by conducting two different tests, i.e. hidden platform acquisition and probe trial test. Briefly, rats were tested in a cylindrical tank of 150 cm in diameter and 60 cm in height. The tank was filled with water until the platform of 12 cm in diameter and 35 cm in height was submerged 1.5 cm below the water surface. The tank is divided into four quadrants with different navigation marks for each quadrant. The midpoint of the wall in each quadrant acts as the starting location of releasing animals into the water.

In the hidden platform acquisition test, the rats were placed in the tank, faced to the wall of the pool, and allowed to freely swim to search for the escape platform within a maximum of 90 s. The platform location remained constant throughout the test. The time of reaching the platform was recorded as the escape latency. The rats were permitted to rest on the platform for 15 s before the next trial. If a rat failed to find the platform within 90 s, it was guided to the platform by a stick and placed on the platform for 15 s; in this case, the escape latency was recorded as 90 s for this trial. The animal was released from a new starting position and the learning trial was repeated until the animal had the desired number of trials. The experiment was repeated with four trials per day and 5 consecutive days. The mean escape latency was measured to evaluate the spatial learning ability.

Twenty-four hours after the hidden platform acquisition test, a probe test was conducted by removing the platform. The rats were placed in the diagonal quadrant of the hidden platform originally located and were allowed to freely swim in the MWM for 60 s. Four indices includingsite crossings (the number of animals crossing the original platform), percent distance and time in target quadrant (the time spent in the target quadrant where the hidden platform was previously located,), and first bearing (animal’s swimming angle at the start of the trial relative to a direct line from the start to the goal) are used to indicate the degree of memory maintenance.

Two Morris water mazes were used by two professional technicians who were blind to the treatments of the animals in this study.

### 4. Determination of Pb concentrations in blood and hippocampus of Wistar rat pups

On PND 21 and PND 91 of the study, blood samples were drawn from the orbitvein and hippocampus tissues were ground in 0.1 M phosphate buffer (pH 7.4) in a mortar under liquid nitrogen. The volume of phosphate buffer is nine times of the weight of hippocampal tissue.

The closed vessel microwave digestion system (CEM-MARS-USA) was used to digest 0.5 ml blood or hippocampal homogenates samples placed in a Teflon digestion vessel with 3 ml of ultrapure HNO_3_ and 1 ml of H_2_O_2_. Sealed containers were placed in a microwave oven and heated according to the digestion program (power, 1600 W (70%); ramp time, 15 min; temperature, 180°C; hold time, 20 min; and cooling time, 15 min). After digestion, sample solutions were cooled to room temperature and then transferred quantitatively into acid cleaned 25-ml standard volumetric flasks and made up to 25 ml with double distilled deionized water. The concentration of lead in blood and hippocampus was measured by an inductively coupled plasma-mass spectrometry (ICP-MS, Agilent 7500c, USA)with the following operating conditions: radio frequency (RF) power 13500 W, RF matching 1.75 V, sample depth 8 mm, carrier gas 0.7 L/min, makeup gas 0.36 L/min, nebulizer pump 0.1 Rps, S/C temp 2°C, and sample analysis time 5 min.

### 5. Nissl staining

The rats on PND 84 were anesthetized with urethane (25%, 0.5 ml/100 g) and intracardially perfused with 0.9% saline, followed by 4% paraformaldehyde in a 0.1 M phosphate buffer (pH 7.4). The brain was carefully removed and post-fixed in 4% paraformaldehyde at 4°C overnight. After standard dehydration and diaphanization procedures, the paraffin-embedded brain was cut into 5-µm thick coronal sections using a rotating microtome (Leica RM2445, Germany). The slides were subjected to Cresyl violet (Nissl) staining. Briefly, the sections were dewaxed, rehydrated, and then immersed in 0.1% Cresyl violet solution at 37°C for 30 min. After being rinsed with double distilled water, they were dehydrated and mounted with permount. Six equidistant sections per brain and 8 different images per region per section were used for cell counting. In each hemisphere, the number of neurons was counted throughout the CA1, CA3, and Dentate gyrus (DG) regions of the hippocampus at 400x magnification (Nikon TE2000-V, Japan) by a person who was blind to the groups of animals via the Imaging-Pro-Plus software 6.0.

### 6. Transmission electron microscopy (TEM)

The rats were anesthetized with urethane before and after MWM and intracardially perfused with 0.9% saline followed by fixative in the mixture of 2% paraformaldehyde and 2.5% glutaraldehyde in a 0.1 M phosphate buffer (pH 7.4) overnight at 4°C.

The sample preparation of TEM was performed as described [14]. Briefly, fixed brains were sectioned coronally at 400 µm on a Leica VT 1000S vibrating microtome. The CA1 region of hippocampus in the sectioned slice was dissected using a stereoscopic microscope, fixed with 1% osmium tetroxide in a 0.1 M phosphate buffer (pH 7.4) for 1 h, dehydrated in a graded series of ethanol solutions and acetone, and embedded with PELCO Eponate 12 kit (Ted Pella, Inc.). Ultrathin sections were cut into 80 nm serially, stained with 4% uranyl acetate and 0.4% lead citrate, and then observed under a Zeiss 10C TEM at 80 kV. Three photos of each sub-region (top, center and bottom) per ultrathin section (3 sections with integrity and sharpness of the structure animal, 10 animals in total per group) were taken at 13,500x and 37,000x magnification, respectively. All the pictures at 13,500x magnification (27 pictures per animal) were used to observe the number of synapses and all the pictures at 37,000x magnification (27 pictures per animal) were used to measure the synaptic structural parameters by selecting typical Gray I synapses with the following characterizations: 1) typical asymmetric interface i.e. the thickness of postsynaptic membrane is much bigger than that of presynaptic membrane; 2) postsynaptic density in the postsynaptic membrane; and 3) round and clear synaptic vesicles [17]. The number of synapses was expressed as the average of all the synapses in each photo taken at 13,500x. The measurement of synaptic cleft, the thickness of postsynaptic density, the length of synaptic active zone and the synaptic curvature was expressed as the average of all synapses in each photo taken at 37,000x as described [18]–[20].

### 7. Statistical analysis

One-way ANOVA, followed by the Bonferroni test, was employed for tests of significance between three or more groups. Wilcoxon Rank-Sum test, a non-parametric test, was used to compare blood and brain Pb concentrations. Results were expressed as means ± S.D. and statistical significance was accepted at *p*<0.05.

## Results

### 1. Developmental Pb exposure enhances blood and hippocampal Pb concentrations in rat pups

The concentrations of Pb in the blood and hippocampal homogenates of rat pups in Pb (pre-weaning) and Pb (post-weaning) groups were significantly higher than those in the control group on PND 21 and 91 (*p*<0.05). It was also found that the blood Pb level was incresed up to above 100 µg/L in Pb (pre-weaning) and Pb (post-weaning) groups, and there was no significant difference between those two groups (*p*>0.05). After weaning, the rat pups of Pb (pre-weaning) group were not exposed to Pb anymore and their blood and hippocampal Pb levels were significantly reduced on PND 91 compared to the group of Pb (post-weaning) (*p*<0.05), but they were still much higher than the group of Control (*p*<0.05) (Table 2).

**Table 2:** Blood and brain hippocampal Pb concentrations in rat pups following lead exposure at different developmental periods.

Group(n = 10)	PND21		PND91	
	Blood Pb (µg/L)	Hippocampal Pb (µg/g)	Blood Pb (µg/L)	Hippocampal Pb (µg/g)
Control	10.09±2.01	0.096±0.002	10.32±2.88	0.104±0.003
Pb(pre-weaning)	103.80±3.25*	0.253±0.008*	39.27±4.17*^#&^	0.196±0.002*^#&^
Pb(pos-tweaning)	-	-	105.45±4.36*	0.261±0.009*

Values are expressed as the means ± S.D. of 10 samples in each group. The symbol of ‘-’ indicates that there is no animal in that group at PND21. * *p*<0. 05, *versus* Control; **^#^** *p*<0. 05, *versus* the group of Pb(post-weaning); **^&^** *p*<0. 05, compared between PND21 and PND91 in each group (Wilcoxon Rank-Sum test).

### 2. Pre-weaning Pb exposure reduces the number of neurons in the hippocampi of rat pups

**Figure 2:**
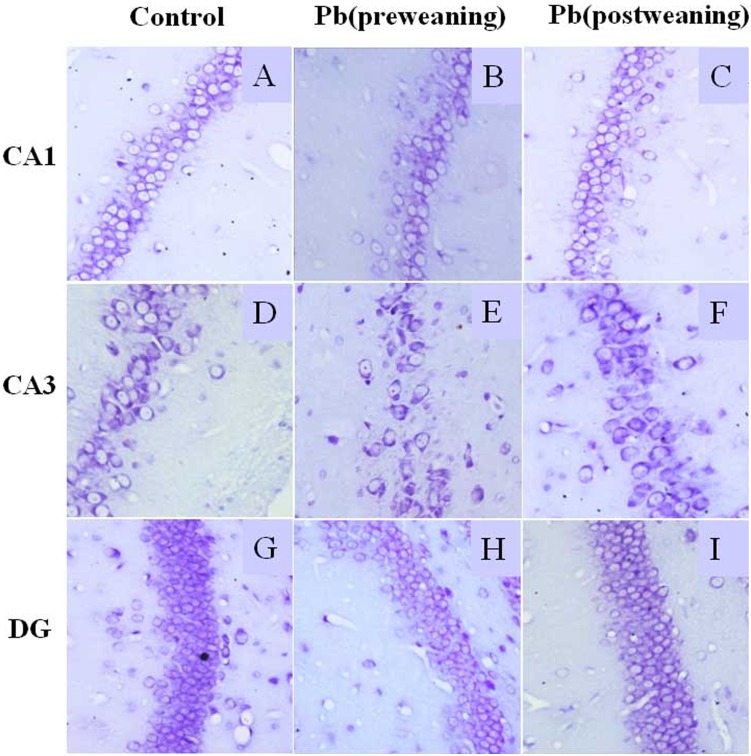
The pre-weaning but not post-weaning Pb exposure reduces the number of neurons in rat pups at postnatal day 90. The representative Nissl staining of neurons nissl bodies in hippocampal subregions (DG, CA1 and CA3) of rat pups from control (A, D, and G), Pb (pre-weaning) (B, E, and H) and Pb (post-weaning) group (C, F, and I). The representative photomicrograph of each region of hippocampus by Nissl staining in each group at 400x magnification. The number of Nissl staining positive neurons was counted as described in Materials and Methods, and calculated as shown in Table 3. The number of neurons in pre-weaning Pb exposure groups was less than that in control group (*p*<0.05).

The Nissl staining of rat hippocampus showed that there were fewer positive neurons in Pb (pre-weaning) group compared to the control group (*p*<0.05, Fig. 2 and Table 3). However, there was no significant difference in neuron numbers between the group of Pb (post-weaning) and the control group (*p*>0.05).

**Table 3:** The effect of developmental lead exposure on the number of neurons in the rat hippocampus on PND 84.

Group (n = 10)	Neuron numbers (400x field)		
	CA1	CA3	DG
Control	67.47±3.81	37.77±3.28	108.57±8.47
Pb(pre-weaning)	42.10±3.39*	26.80±2.86*	78.32±7.94**
Pb(post-weaning)	66.17±3.74	39.75±3.55	107.13±10.34

Values are expressed as the means ± S.D. of 10 samples in each group; * *p*<0. 05 and ** *p*<0. 01, *versus* Control (one-way ANOVA and the Bonferroni test).

### 3. Effects of developmental Pb exposure on the number of synapses in the CA1 regions of rat pups

The quantitative data of the number of synapses (Table 4) showed that, before the MWM training, pre-weaning Pb exposure significantly reduced the number of synapses (*p*<0.05), whereas there was no significant difference in the synapse number between Pb (post-weaning) group and the control group (*p*>0.05). Following the MWM training, the number of synapses in the control group and Pb (pre-weaning) group increased significantly, although the number in Pb (pre-weaning) group was still significantly less than that in the control group. But there was no significant increase in the number of synapses in Pb (post-weaning) group. In addition, the fold-increase of the number of synapses before and after the MWM training in Pb (pre-weaning) group is similar to that in the control group.

**Table 4:** The effect of developmental lead exposure on the number of synapses in the CA1 region of rat hippocampus.

Group (n = 10)	Synapse numbers (13500x field)	
	Before MWM (PND84)	After MWM (PND91)
Control	30.01±2.49	41.24±3.77^#^
Pb(pre-weaning)	19.32±3.78*	26.80±4.26*^#^
Pb(post-weaning)	29.55±3.44	34.09±4.31*

Values are expressed as the means ± S.D. of 10 samples in each group; *p<0. 05, versus Control (one-way ANOVA and the Bonferroni test);^ #^ *p*<0. 05, compared between Before MWM and After MWM in each group (t test).

### 4. Developmental Pb exposure alters the synaptic structural parameters in the CA1 regions of rat pups

**Figure 3:**
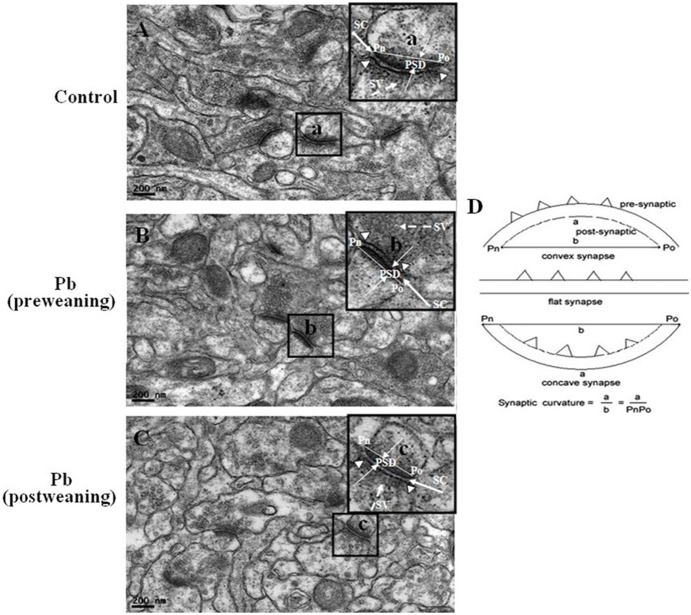
The synaptic structure in the CA1 region of rat hippocampus at postnatal day 90 was observed under transmission electron microscopy (37,000x). The synaptic structural parameters were measured as described in the section of Materials and Methods. Also, the synaptic structural parameters of control (A), Pb (pre-weaning) (B), and Pb (post-weaning) group (C) were compared in Table 5. The ultrastructural features of synapses (insets a–c) in three groups (A, B, C) and the synaptic curvature measurement (D) were illustrated. The length of synaptic active zone was indicated by two arrowheads. The thickness of postsynaptic density (PSD) was at the thickest part of PSD and limited by two long arrowheads; Po and Pn showed the synaptic curvature measurement points; SC represents the synaptic cleft; SV represents synaptic vesicles.

During the process of learning and memory, the synaptic cleft decrease, while the thickness of postsynaptic density, the length of synaptic active zone and the synaptic curvature increase. The measurement of synaptic structural parameters in the representative series of electron micrographs (Fig. 3) showed that the synaptic cleft in Pb (pre-weaning) or Pb (post-weaning) group was significantly wider than that in the control group, and the thickness of postsynaptic density, the length of synaptic active zone, and the synaptic curvature were significantly smaller than those in the control group (*p*<0.05) (Table 5). These results suggest that both early and later developmental Pb exposure can impair the synaptic structure in the CA1 regions of rat pups.

**Table 5:** The effect of developmental lead exposure on the synaptic structure parameters in the CA1 region of rat hippocampus on PND91.

Group(n = 10)	Synaptic cleft (nm)	PSD thickness (nm)	Length of synaptic active zone (nm)	Synaptic curvature
Control	17.41±0.45	76.72±3.40	244.07±6.43	1.401±0.027
Pb(pre-weaning)	21.77±0.58*	63.24±3.08*	211.65±8.38*	1.062±0.042*
Pb(post-weaning)	21.25±0.62*	61.55±5.44*	220.50±7.64*	1.109±0.044*

Values are expressed as the means ± S.D. of 10 samples in each group; * *p*<0. 05, *versus* Control (one-way ANOVA and the Bonferroni test).

### 5. Developmental Pb exposure impairs the spatial learning and memory in rat pups

**Figure 4:**
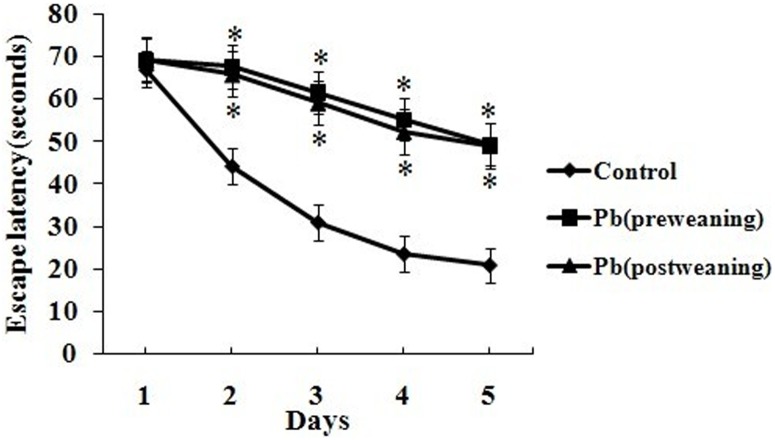
The mean escapes latency of rat pups with different Pb exposures in the 5-day learning trials of MWM test. Data are expressed as the mean ± S.D. of the mean escape latency of 10 animals in each group; *p*<0.05, versus control (One-way ANOVA followed by the Bonferroni test).

The spatial learning and memory was measured by MWM test as described in the Materials and Methods. The results (Fig. 4) showed that the mean escape latency decreased gradually day by day in all the groups, but the mean escape latency of Day 2–5 increased significantly (*p*<0.05) in both Pb (pre-weaning) and Pb (post-weaning) groups, compared to the control group. There was no significant difference in the swimming speed in each group of rats (data not shown). These results indicate that both early and late developmental Pb exposure will impair the learning ability in MWM test. In the probe trial, decreased site crossings and increased percent distance in target quadrant, percent time in target quadrant and first bearing we found in both Pb (pre-weaning) and Pb (post-weaning) groups, compared to the control group (*p*<0.05), suggesting that both early and late developmental Pb exposure will impair the spatial memory ability in the MWM test (Table 6).

**Table 6:** Effect of developmental lead exposure on the performance of rat pups in the probe trial of MWM test on PND85–90.

Group(n = 10)	Sitecrossings	Percent distance intarget quadrant (%)	Percent time intarget quadrant (%)	First bearing (^o^)
Control	3.80±0.35	32.60±1.4	32.70±2.56	8.74±1.44
Pb(pre weaning)	1.48±0.42**	17.35±1.09***	17.79±1.97**	28.68±3.43***
Pb(post weaning)	1.38±0.35**	16.79±1.90***	17.15±2.26**	27.99±3.17***

Values are expressed as the means ± S.D. of 10 samples in each group; ** *p*<0. 01, *** *p*<0.0 01, *versus* Control (one-way ANOVA and the Bonferroni test).

## Discussion

During the early developmental stage of CNS, neurogenesis is the self-proliferation of neural progenitor cells and their differentiation into neuronal cells. In the study of the directed differentiation of human embryonic stem cells, it was found that cultured neural progenitor cells mostly differentiated into neurons instead of glia during early passages. The percentage of glia, however, increased significantly as the culture time extended [21]. Pb exposure at low level (0.01–10 µM) caused a significant dose-dependent inhibition of proliferation (assessed by ^3^H-thymidine uptake) of neural stem cells (NSCs) originating from rat ventral mesencephalon (VM) and striatum (ST), and decreased the number of MAP2 positive neurons differentiated from NSCs originating from those regions [22]. Chronic developmental lead (0.2% Pb-acetate) exposure reduced neurogenesis in the dentate gyrus (DG) of adult rat hippocampus [15]. Prenatal and neonatal Pb (0.1% Pb-acetate) exposure reduced the number of neurons in the CA1 region of rat hippocampus [23]. In this study, we also found that pre-weaning Pb exposure reduced the number of neurons in the DG as well as in other regions of rat hippocampus. These results suggest that Pb exposure may inhibit the proliferation of neural progenitor cells as well as their differentiation of neurons at the early developmental stage.

During the late developmental stage of CNS, the differentiation of neural progenitor cells into neurons decreases significantly. Most of newly generated cells are glia and the number of neurons relatively keeps stable during the period of weaning. Following the maturation, neural progenitor cells usually stay at a resting state without proliferation unless they are stimulated under certain conditions and mostly they are directed to differentiate into glia instead of neurons. Also, differentiated neurons cannot divide anymore once they are generated [24], [25]. Most of previous studies were performed in animals exposed to Pb at the early stage of development starting from gestation and/or lactation, resulting in the inhibition of neurogenesis and differentiation of hippocampal neurons in young adult animals [15], [26], [27]. However, none of them have investigated whether the effects of Pb on adult neurogenesis and neuronal differentiation are specific to a defined stage of development. We found in the present study that Pb exposure after the weaning did not affect the number of neurons in the hippocampus of rat pups, suggesting that the late developmental Pb exposure (e.g. post-weaning) might not inhibit the neurogenesis and differentiation of hippocampal neurons in young adult rats.

Synapse is a highly specialized connection between two neurons and is essential for the functional connection and signal transmission between neurons [28]. The gradual formation of synapse is associated with the differentiation and migration of neurons [29], [30]. The embryonic Pb exposure might inhibit the differentiation of neurons as well as the formation of synapses at the same time. The already formed synapses, however, might not be damaged by Pb exposure after the completion of differentiation and migration of neurons. This was evidenced by our findings that the number of synapses in the CA1 region of rat hippocampus both before and after the MWM training was significantly reduced by pre-weaning Pb exposure compared to that in the control rat pups (*p*<0.05), whereas the number of synapses before the Morris water maze training was not altered by post-weaning Pb exposure in rat pups (*p*>0.05). It should be noted that the number of synapses in Pb (pre-weaning) group increased significantly following MWM training (*p*<0.05), even with the similar percentage of increase to that of the control group. The spatial learning and memory ability, however, did not show any significant improvement in Pb (pre-weaning) group. This might be due to the fact that a significant loss of neurons and synapses already happened in rat pups exposed to Pb before weaning. Although the number of synapses in rat pups of Pb (pre-weaning) group increased significantly following MWM training (*p*<0.05), it was still significantly less than that in the control rat pups (*p*<0.05). The increase of synapse numbers may not be able to compensate for the learning and memory deficits caused by the significant loss of neurons and synapses already happened in rat pups exposed to Pb before weaning.

Postsynaptic density (PSD) is a most important structural basis for synaptic structural plasticity and its thickness, length and area was found to increase during the induction of LTP [14]. The interface connecting the presynaptic and postsynaptic components of synapses is not always flat and mostly has a certain curvature, which is named synaptic curvature. No matter during the development or maturation, the change of synaptic shape is always associated with synaptic functions [31]. The curvature of synaptic connection interface is a way to enhance the area of connection, which ensures the delivered neurotransmitters get to the target instead of diffusing to the peripheral spaces, enhancing the transmitting efficacy of those transmitters. Furthermore, alterations in above synaptic structural parameters may be regulated by neural cell adhesion molecule (NCAM). At the resting state, NCAM undergoes posttranslational modifications that involve adding several α-2,8-polysialic acid (PSA) residues in its extracellular domains, which is beneficial for keeping the structural stability of synapses. During the process of learning and memory, the nerve impulse will remove those PSA residues from NCAM so that astrocytes surrounding the synapses will shrink because of the change of their adhesion strength, resulting in the decrease of synaptic cleft and the increase of PSD, the length of synaptic active zone and the synaptic curvature [32]–[36]. We have previously reported that maternal low-level Pb exposure reduced the expression of PSA-NCAM and the activity of sialyltransferase in the hippocampus of neonatal rat pups [16], which might affect the synaptic structure during development. In this study, we verified that developmental Pb exposure altered the number of synapses and the synaptic structural parameters. These results suggest that developmental Pb exposure might affect the synaptic structures via reducing the adhesive ability of NCAM. The alterations of synaptic structure such as increase of synaptic cleft and the decrease of PSD, the length of synaptic active zone and the synaptic curvature can increase the distance between presynaptic membrane and postsynaptic membrane and reduce their contact area, resulting in the decrease of transmitting efficacy of neurotransmitter. The decrease of synaptic transmission efficiency will block the synaptic potential formation and impair of the ability of learning and memory. The population investigation found that there were significant associations between prenatal/postnatal blood lead levels and the cognitive development of children [37], [38]. Both clinical and preclinical studies indicate that the developing brain is particular sensitive to pernicious effects of Pb exposure. It has been found that Pb exposure significantly increases neuronal apoptosis in neonatal mice on PND 7, indicating that the immature nervous system may be particularly susceptible to Pb exposure [39]. Low-level Pb exposure during the gestational period significantly impairs the spatial learning and memory of young adult offspring [40]. Furthermore, Pb exposure at weaning still produced learning and memory deficits in adult rats [41], [42]. In the present study, we found that both pre weaning and post weaning Pb exposure impaired the spatial learning and memory. Based on the results mentioned above, we found that the fold-increase in the number of synapses after the MWM training was much higher in the pre-weaning Pb group (44% increase) than in the post-weaning Pb group (13% increase) (Table 4), yet the synaptic structure was similarly impaired both in pre- weaning and post-weaning Pb group (Table 5). It seems that the effects of Pb on the new synaptic formation and the synaptic structural parameters are differed depending on the Pb exposure time.

Interestingly, in our another study that focusing on the protective role of selenium on Pb-induced neurotoxicity, we demonstrated that organic Selenium significantly ameliorated the impairments of spatial learning and memory as well as synaptic structural plasticity induced by Pb exposure in rats after weaning, but not by the maternal Pb exposure which reduced the numbers of neurons and synapses in the early neural development [43]. These findings, from another perspective, strengthen the role of synaptic structural plasticity in impairments of spatial learning and memory induced by developmental lead exposure in Wistar rats, which is the focus of this study. Also, our previous findings suggest that the cognitive impairments induced by early developmental Pb exposure might be difficult to restore.

In summary, we found that late developmental Pb exposure (e.g. post weaning) affected the synaptic structure as well as the regeneration of synapses to impair the learning and memory of rat pups. The early developmental Pb exposure (e.g. pre weaning) not only affected the synaptic structure but also reduced the number of differentiated neurons and synapses significantly, resulting in the inhibition of spatial learning and memory. Since the number of neurons and synapses were uncompensated in our study, the cognitive impairment induced by early developmental Pb exposure might be difficult to restore, which warrants further long-term experiments to verify. Our data suggest that the keystone of the prevention and treatment of Pb intoxication should not only focus on developing children but also pay more attention to women before and during gestation.
